# A serologic study of dengue in northwest Ethiopia: Suggesting preventive and control measures

**DOI:** 10.1371/journal.pntd.0006430

**Published:** 2018-05-31

**Authors:** Getachew Ferede, Moges Tiruneh, Ebba Abate, Yitayih Wondimeneh, Demekech Damtie, Endalamaw Gadisa, Rawleigh Howe, Abraham Aseffa, Belay Tessema

**Affiliations:** 1 Department of Medical Microbiology, School of Biomedical and Laboratory Sciences, College of Medicine and Health Sciences, University of Gondar, Gondar, Ethiopia; 2 Ethiopian Public Health Institute, Addis Ababa, Ethiopia; 3 Armauer Hansen Research Institute, Addis Ababa, Ethiopia; CDC, UNITED STATES

## Abstract

**Background:**

Dengue is one of the most serious and rapidly spreading arboviral diseases in the world. Despite many acute febrile illnesses in Ethiopia, the burden of illness due to dengue in the country is largely unknown. Thus, the present study aimed to provide the first baseline data on seroprevalence and associated risk factors of dengue virus (DENV) infection in the country.

**Methods:**

A cross-sectional study of febrile patients who were visiting Metema and Humera hospitals in Northwest Ethiopia from March 2016 to May 2017 was conducted. Blood samples were collected from each participant and serum samples were separated and tested for IgM and IgG antibodies against DENV infection by enzyme-linked immunosorbent assay (ELISA). Risk factors associated with the prevalence of anti-DENV antibodies were tested using logistic regression analysis.

**Results:**

Of the 600 samples tested, the overall seroprevalence against DENV infection was 33.3%, while the seroprevalence by the study area was 40% in Metema and 27.5% in Humera. The overall prevalence of IgM and IgG antibodies against DENV infection was 19% and 21% respectively. Of these, 6.7% were positive for both IgM and IgG antibodies. Residence and occupational status were significantly associated with the prevalence of anti-DENV IgM seropositivity and anti-DENV IgM-/G+serostatus. The seasonal variation was significantly associated with the prevalence of anti-DENV IgM but not with anti-DENV IgM-/G+serostatus. The prevalence of anti-DENV IgM-/G+serostatus was significantly higher in Metema than Humera. High prevalence of anti-DENV IgM seropositivity was found in the summer and spring, with a peak in the month of August. The presence of uncovered water either indoor or outdoor and lack of mosquito net use was identified as risk factors for DENV infection.

**Conclusions:**

These findings provide the preliminary data on seroprevalence and associated risk factors of DENV infection in the country. The presence of antibodies against DENV infection indicates dengue as one of the causes of undifferentiated febrile illnesses in the study areas. This suggests that prevention and control measures should be designed considering the risk factors identified by this study. Furthermore, we recommend a large-scale study to include DENV infection in the differential diagnosis of all febrile illnesses in Ethiopia.

## Author summary

Despite dengue is currently one of the leading causes of arboviral diseases in the globe, it is unrecognized and underreported in Africa, particularly in Ethiopia. Thus, we conducted a cross-sectional study among febrile patients who were attending health institutions to document seroprevalence and associated risk factors of DENV infection in the country. The study illustrated the presence of antibodies against DENV infection for the first time in both study areas, an awaking message for those who were involved in health sectors. Most of the active DENV transmission was found in monsoon and post-monsoon periods with a peak in the month of August. In multivariate analysis residence, occupational status and seasonal variations were significantly associated with the prevalence of anti-DENV IgM seropositivity. Moreover, individuals who lack a mosquito net use and the presence of uncovered water storages either indoors or outdoors were identified as the risk factors of DENV infection. Therefore, we recommend that preventive measures should be considered. Moreover, nationwide surveillance should be carried out at large.

## Introduction

Dengue is one of the most important mosquito-borne viral diseases and can be caused by any one of the four dengue virus serotypes (DENV1-4) of the genus Flavivirus [1]. Dengue virus is a non-segmented, positive-sense, single-stranded, enveloped RNA virus; transmitted mainly by the bite of *Aedes aegypti*, a tropical and subtropical mosquito species that inhabits mostly in urban areas in proximity to houses [2]. It breeds in small bodies of fresh water, most commonly in various containers found around homes [3]. It is estimated that 390 million DENV infections occur every year [4], and it is endemic in more than 100 countries across the Americas, East Mediterranean, Western Pacific, Africa and South-East Asia and Europe [5]. In Africa, dengue has been reported in 34 countries, mostly in Eastern Africa [6]. In the countries bordering Ethiopia such as Sudan, Eritrea, Kenya and Djibouti dengue has also been reported [7–11]. Four of the DENV serotypes have been detected in Africa, with the DENV-2 serotype reported to cause the majority of epidemics [6]. The DENV-2 serotype and the main dengue vector, *Ae*. *aegyptus* were recently reported in Dire Dawa Ethiopia [12]; even prior to the DENV outbreak the presence of *Ae*. *aegypti* in different regions of Ethiopia had been reported [13, 14].

Although dengue has a global distribution, the vast majority of the data on DENV infections are from the WHO South-East Asia and Western Pacific Regions [2]. In Ethiopia, prior to 2013, there were no reports of dengue. However, recently, DENV infection has emerged in Dire Dawa and Somalia regions [12, 15]. In the present decade, dengue has expanded into new countries where it had not existed earlier [2]. The expansion of dengue is expected to increase due to several factors such as human population growth, climate change, and increased urbanization with sub-standard housing, irregular water supply, and poor environmental sanitation. Together with increased mobility of both vectors and human populations all over the world, further spread of dengue from the endemic areas to many previously unaffected areas is anticipated [16].

Dengue is an acute febrile illness, which occurs after an incubation period of 4–10 days. Dengue disease severity varies from asymptomatic infection to a variety of illnesses ranging from an influenza-like self-limiting illness to a potentially lethal disease such as dengue hemorrhagic fever (DHF) or dengue shock syndrome (DSS) [17, 18]. It is a complex disease with various clinical presentations, which often go unrecognized or misdiagnosed as other common fever-causing tropical diseases. For instance, malaria is endemic throughout the African region including Ethiopia; the majority of the febrile illnesses including dengue, are likely to be misdiagnosed and treated as presumptive malaria [6]. Because of this, dengue can progress from a mild, nonspecific viral disease to severe disease. Without good clinical management, mortality from the complications of DHF/DSS can be as high as 20 percent, whereas if the case is recognized early and properly managed, mortality due to the complications declines to less than 1 percent [19, 20]. Hence, early and rapid laboratory diagnosis of dengue is important for proper management and prevention of complications like DHF/DSS [20, 21].

Studies in Ethiopia have indicated that the unknown causes of acute febrile illnesses are high [22]. Among the causes of non-malaria febrile diseases around the globe, DENV infection is currently considered as the leading cause of febrile illness [23]. However, data are not available on the prevalence of dengue in Northwest Ethiopia. Considering the current situation of DENV infection and unavailability of data in the country, this study was therefore conducted to document the first baseline seroprevalence and risk factors associated with DENV infection in the country. The present study will be helpful in providing information on DENV infection to healthcare authorities for better clinical management of patients and to design and implement appropriate control measures.

## Materials and methods

### Ethics statement

The study was reviewed and approved by the Ethical Review Committees of the University of Gondar and Armauer Hansen Research Institute. The study participants were informed about the study before collecting any data or samples. Written informed consent was obtained from each participant or assent from each parent/guardian of the children. Blood samples were collected by experienced laboratory technologist as part of the routine sample collection. Participants had full right to continue or withdraw from the study. The confidentiality of all participants was maintained throughout the study.

### Study area

**Fig 1:**
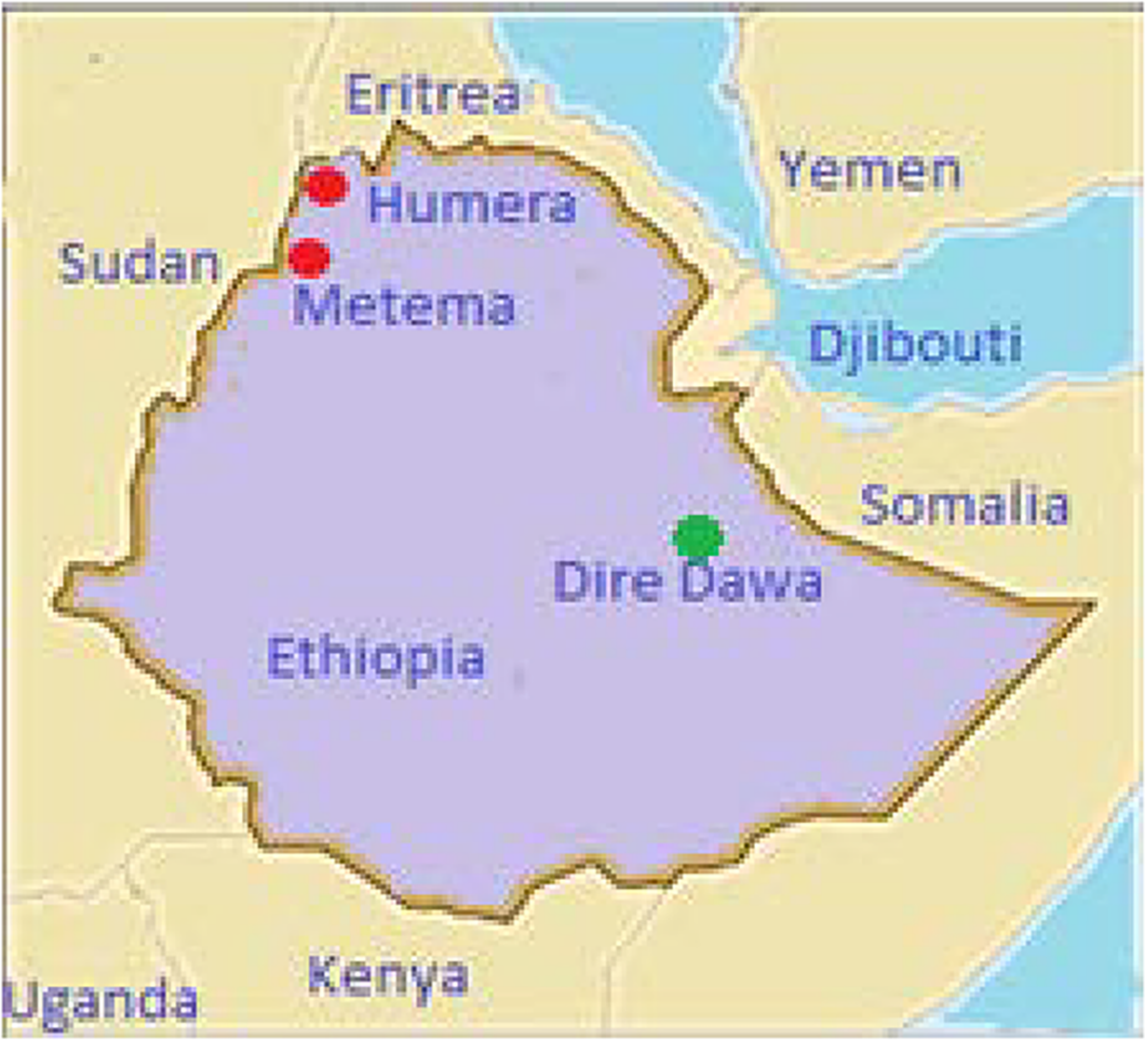
Dengue study areas-Humera and Metema hospital, and other recent outbreaks in Dire Dawa town. Red: current dengue sero-study areas; green: recent dengue reported area in Ethiopia.

The study was carried out in Northwest Ethiopia; Metema and Humera Kahsay Abera hospitals (Fig 1). The Metema hospital is located in Northwest Ethiopia on the border with Sudan, 897 km North of Addis Ababa and 197 km from the ancient city of Gondar. This town has a latitude and longitude of 12°58′N 36°12′E with an elevation of 685 m above sea level. The Humera Kahsay Abera hospital is also located in Northwest Ethiopia, 252 km from Gondar city and 974 km from Addis Ababa and located in the western zone of the Tigray Regional state, bordered on the west by Sudan, and on the north by the Tekezé River which separates Ethiopia from Eritrea. This town has a latitude and a longitude of 14° 18’N36° 37’E with an elevation of 602 m above sea level. Both Metema and Humera areas are one of the hottest and malaria-endemic areas in the country and the most fertile agricultural zones with the large scale of farming of cash crops.

### Study design, period and patient’s characteristics

A cross-sectional hospital-based study was carried out among febrile patients attending the two hospitals from March 2016 to May 2017. The sample size was estimated to be 600 by using single proportion formula at 95% confidence interval, an expected prevalence of 50% and 4% marginal error. The study participants were all febrile patients who have been presumed for DENV infection. Dengue was presumed in patients who live or traveled to an endemic area and presented with fever and two of the following criteria such as nausea or vomiting, rash, aches and pains, tourniquet test positive, leukopenia, and any warning sign based on WHO-TRD 2009 criteria; further suggested dengue cases based on the levels of severity were classified as dengue without warning signs, dengue with warning signs, and severe dengue. Dengue without warning signs was defined as laboratory-confirmed dengue cases without signs of plasma leakage. Dengue with warning signs included abdominal pain or tenderness, clinical fluid accumulation, persistent vomiting, lethargy, restlessness, mucosal bleeding, liver enlargement >2 cm, an increase in hematocrit (HCT) concurrent with a rapid decrease in platelet count. Severe dengue; in addition to aforementioned criteria, includes signs of severing plasma leakage and/or severe bleeding, severe organ impairment [2]. A febrile patient was defined as a patient who came to either the outpatient or inpatient department and to either the pediatric or medicine unit at the participating hospital with fever ≥ 38°C. Prevalence of DENV infection was defined as the proportion of participants with IgM and /or IgG ELISA positive.

### Data collection

Study participants who met the case definition were screened by clinicians. When a participant was willing to participate in the study, clinical features, demographic information, and risk factors were collected by a nurse using a structured questionnaire that underwent validation and editing after small pilot study. A 3 to 5ml of blood was collected from each eligible study participant by venepuncture into a new sterile plain test tube to obtain serum. The blood samples were centrifuged for 5 min, aliquoted and stored at –20°C until processed. Serum samples were tested for IgM and IgG antibodies to DENV with ELISA (EUROIMMUN). The anti-DENV antibodies (IgM and IgG) ELISA were performed as per the manufacturer's instructions [24].

### Data analysis

Data were entered and analyzed using SPSS version 20 software. Simple frequency tables were generated, and categorical variables were compared using chi-square test. A univariate logistic regression analysis was used to identify risk factors associated with the prevalence of anti-DENV IgM and IgG antibodies. Those independent variables found p < 0.2 in univariate analysis were then used in multivariate logistic regression analysis. Odds ratios (ORs) at 95% confidence intervals (CIs) were calculated to measure the degree of association. A p-value < 0.05 was considered as statistically significant and data were presented in the form of tables and figures.

## Results

### Socio-demographic characteristics

All the study participants who have fulfilled the inclusion criteria of the study were enrolled and completed the study. A total of 600 febrile patients who were presumed for DENV infection were enrolled in this study. Of these, 394 (65.7%) of the participants were males and the mean age of the participants was 25 years, ranging from 1 to 78 years. The majority of the participants 302 (50.3%) were between 15 to 29 years of age groups, while the 68 (11.3%) were greater or equal to 45 years of age groups. Three hundred sixteen (52.7%) study participants were urban dwellers. Most of the participants 301 (50.2%) were farmers, followed by 165 (27.5%) students. Among the study participants, 184 (30.7%) were illiterate (Table 1).

**Table 1:** Socio-demographic characteristics of the study participants, from March 2016 to May 2017.

Variable	Frequency	Percentage
**Age (years)**		
≤ 14	116	19.3
15–29	302	50.3
30–44	114	19.0
≥ 45	68	11.3
**Gender**		
Male	394	65.7
Female	206	34.3
**Residence**		
Urban	316	52.7
Rural	284	47.3
**Marital status**		
Married	255	42.5
Single	332	55.3
Divorced	13	2.2
**Occupation**		
Farmer	301	50.2
Shopkeeper	48	8.0
Government employee	34	5.7
Day laborer	38	6.3
Student	165	27.5
Not applicable	14	2.3
**Educational status**		
Illiterate	184	30.7
Only read and write	145	24.2
Elementary school	170	28.3
Secondary school	77	12.8
Higher education	24	4.0

### Seroprevalence of DENV infection

Of the 600 study participants screened for DENV specific IgM and IgG antibodies, 114 (19%) were positive for IgM and 126 (21%) for IgG and 40 (6.7%) samples were positive for both IgM and IgG (Table 2). This showed a total of 200 (33.3%) positive cases for IgM/G antibodies. Of the total study participants, 74 (12.3%) study participants had IgM+/G- and 86 (14.3%) had IgM-/G+ serostatus. As per WHO-TDR (Tropical Diseases Research) 2009 guidelines (2); among the 114 anti-DENV IgM seropositive patients, 84 (73.7%) were classified as dengue without warning signs, 30 (26.3%) as dengue with warning signs, and none of the patients were classified as severe dengue (Table 3).

**Table 2:** Laboratory diagnosis of anti-DENV IgM and IgG antibodies using ELISA from March 2016 to May 2017.

Study area	Total no. of participant	Antibodies against DENV infection		
		IgM against DENV (%)	IgG against DENV (%)	Both IgM and IgG against DENV (%)
Humera Kahsay Abera hospital	320	54 (16.9)	51 (15.9)	17 (5.3)
Metema hospital	280	60 (21.4)	75 (26.8)	23 (8.2)
Total	600	114 (19)	126 (21)	40 (6.7)

**Table 3:** Suggested dengue case classification based on WHO-TDR 2009 criteria.

Classification	Frequency	Percentage
Dengue without warning signs	84	73.7
Dengue with warning signs	30	26.3
Total	114	100

**Fig 2:**
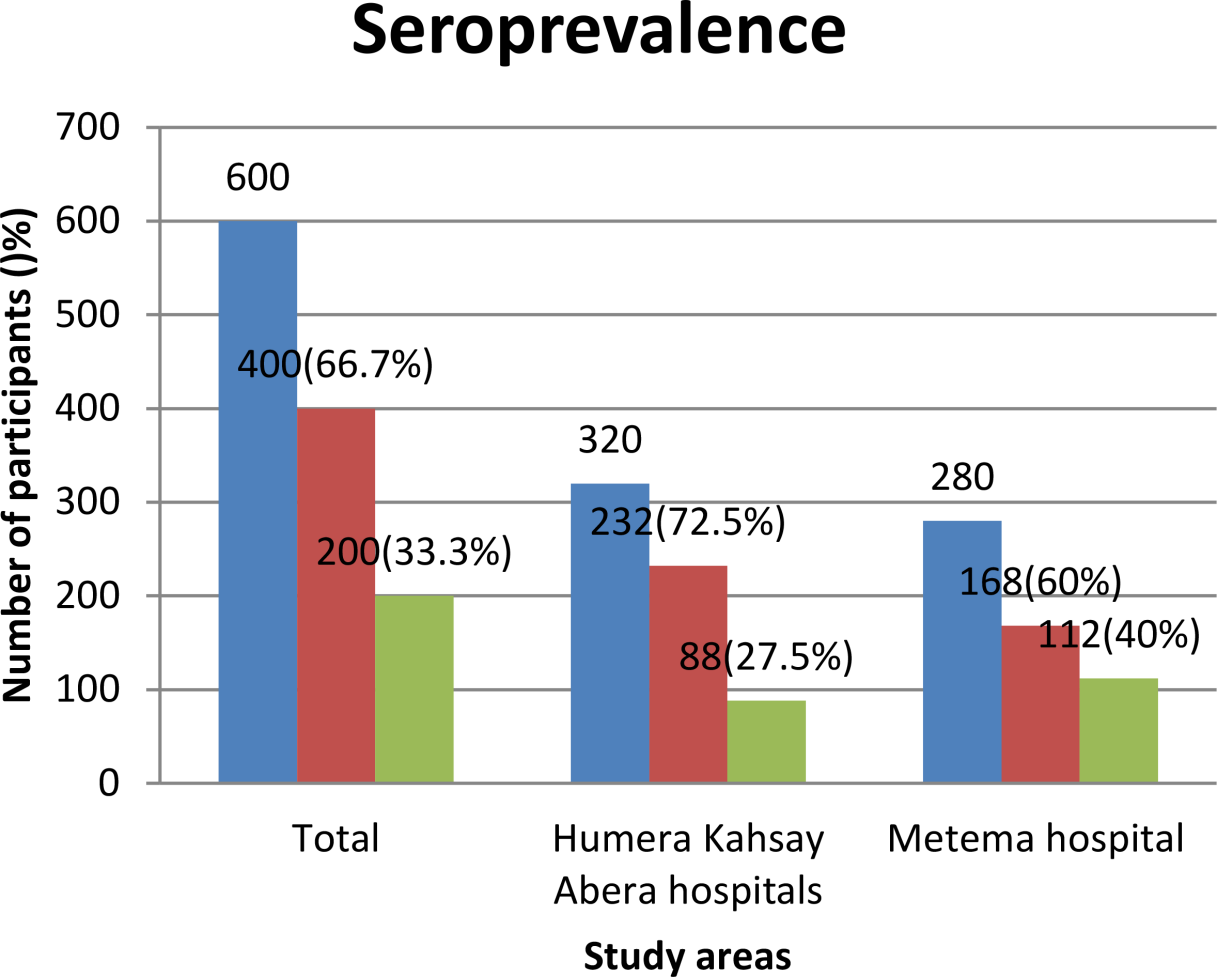
Seroprevalence of DENV infection, from March 2016 to May 2017. Blue: total number of study participants; red: study participants negative against anti-DENV antibodies; green: study participants positive against anti-DENV antibodies.

Out of 600 study participants, the overall seroprevalence of DENV infection was 33.3% (200/600; 95%CI, 29.7–37.3). Of this, Metema accounts 56% (112/200) and Humera 44% (88/200) of the cases. The seroprevalence by study area was 40% (112/280) in Metema hospital and 27.5% (88/320) in Humera Kahsay Abera hospital (Fig 2). None of the study participants had been vaccinated against yellow fever.

**Fig 3:**
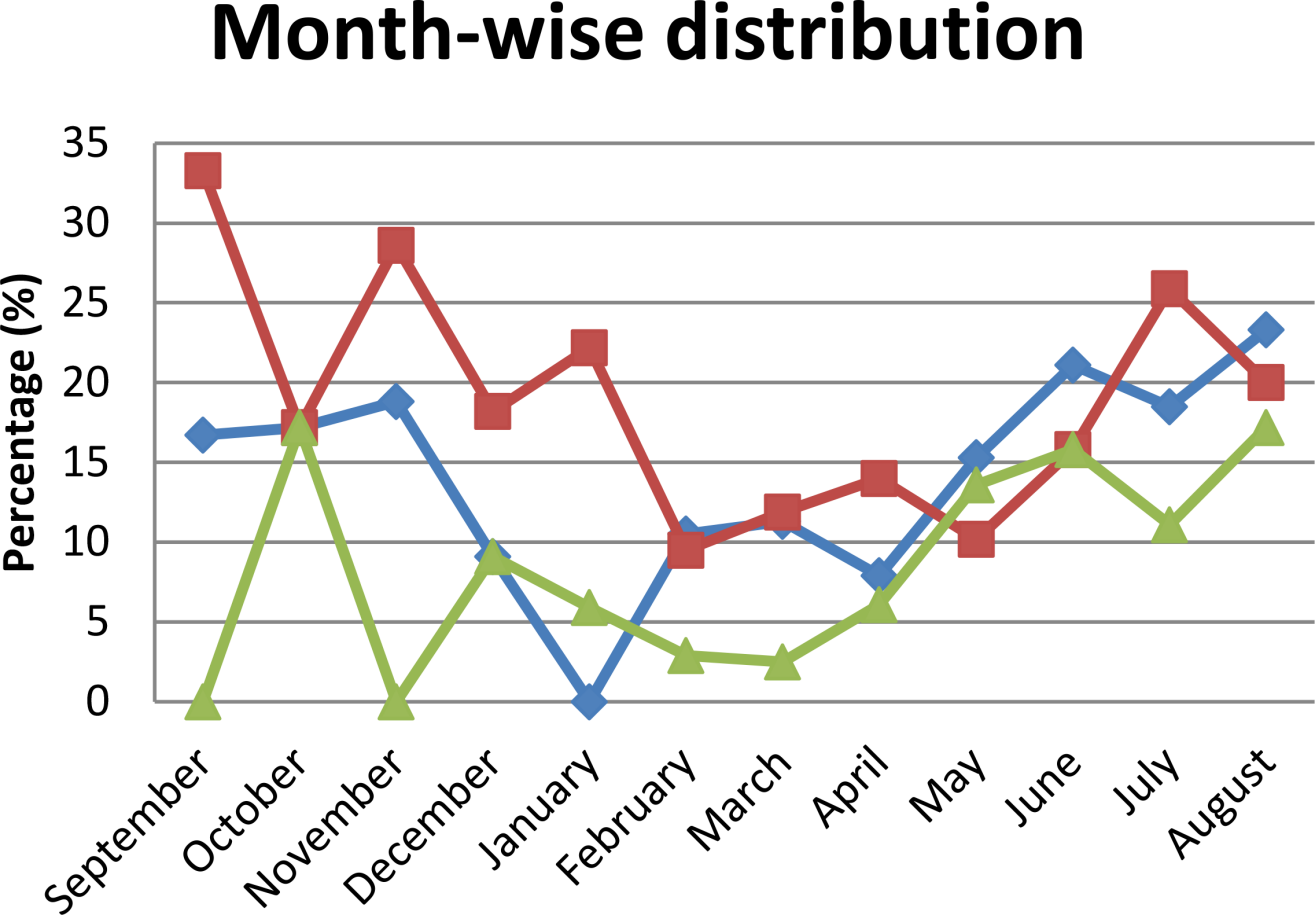
Month-wise distributions of antibodies against DENV infection, from March 2016 to May 2017. Blue diamonds: study participants with only anti-DENV IgM positive cases; red squares: study participants with only anti-DENV IgG positive cases; green triangles: study participants with both IgM and IgG positive cases.

The month-wise distribution of anti-DENV antibodies showed a high proportion of only IgM in June to November with the peak in the month of August, which is conceded with the monsoon and post-monsoon periods while from December to May its prevalence was law and irregular. The prevalence’s of both anti-DENV IgM and IgG antibodies were irregular with high prevalence in the months of August and October. Seropositivity of only anti-DENV IgM was detected in almost every month of the year except January while only anti-DENV IgG was detected in each month of the year. Seropositivity of both anti-DENV IgM and IgG antibodies were detected in every month of the year except September and November (Fig 3).

### Associated factors with the seroprevalence of anti-DENV antibodies

In a univariate analysis, gender, residence, occupational status and season showed statistically significant association with the prevalence of anti-DENV IgM seropositivity (p-value < 0.05) while age, study area, the marital and educational status of the study participants were not significantly associated. In multivariate analysis, occupational status and season remained significantly associated with the prevalence of anti-DENV IgM seropositivity. Those study participants who lived in urban areas were 1.8 times (AOR = 1.85; 95% CI = 1.18–2.89) more likely to have anti-DENV IgM seropositivity than those who lived in rural areas; farmers were 2 times (AOR = 2.03; 95% CI = 1.14–3.61) more likely to have anti-DENV IgM seropositivity than the students. During summer; June to August, 3.2 times (AOR = 3.22; 95%CI = 1.58–6.56) higher anti-DENV IgM seropositivity was observed, followed by spring; September to November (which is the monsoon and post-monsoon period in Ethiopia) than winter; December to February (Table 4).

**Table 4:** Factors associated with the prevalence of anti-DENV IgM seropositivity among the study participants, from March 2016 to May 2017.

Variable	Total No. tested (N = 600)	Anti-DENV IgM+	A univariate analysis	Multivariate analysis	MP-value
		N (%) (N = 114)	COR (95%CI)	AOR (95%CI)	
**Age**					
≤ 14	116	20 (17.2)	1		
15–29	302	53 (17.5)	1.02 (0.58–1.79)		
30–44	114	26 (22.8)	1.41 (0.74–2.71)		
≥ 45	68	15 (22.1)	1.35 (0.64–2.87)		
**Gender**					
Male	394	88 (22.3)	1.99 (1.23–3.20)*	1.61 (0.97–2.66)	0.061
Female	206	26 (12.6)	1	1	
**Residence**					
Urban	316	71 (22.5)	1.62 (1.06–2.46)*	1.85 (1.18–2.89)*	0.007
Rural	284	43 (15.1)	1	1	
**Marital status**					
Married	255	46 (18)	1.21 (0.25–5.64)		
Single	332	66 (19.9)	1.36 (0.29–6.30)		
Divorced	13	2 (15.4)	1		
**Occupation**					
Farmer	301	71 (23.6)	2.23 (1.30–3.83)*	2.03 (1.14–3.61)*	0.016
Not applicable	14	3 (21.4)	1.97 (0.50–7.70)	1.96 (0.47–8.16)	
Shopkeeper	48	6 (12.5)	1.03 (0.39–2.74)	1.01 (0.36–2.80)	
Government employee	34	8 (23.5)	2.23 (0.88–5.59)	1.93 (0.75–4.96)	
Day laborer	38	6 (15.8)	1.35 (0.50–3.65)	1.21 (0.43–3.36)	
Student	165	20 (12.1)	1	1	
**Educational status**					
Illiterate	184	38 (20.7)	1.30 (0.42–4.03)		
Only read and write	145	27 (18.6)	1.14 (0.36–3.62)		
Elementary school	170	32 (18.8)	1.15 (0.37–3.62)		
Secondary	77	13 (16.9)	1.01 (0.29–3.46)		
Higher education	24	4 (16.7)	1		
**Study area**					
Humera	320	54 (16.9)	1		
Metema	280	60 (21.4)	1.34 (0.89–2.02)		
**Season**					
Summer	77	28 (36.4)	3.93 (1.97–7.83)*	3.22 (1.58–6.56)*	0.001
Spring	56	14 (25)	2.29 (1.04–5.05)*	1.81 (0.80–4.07)	
Autumn	333	55 (16.5)	1.36 (0.75–2.44)	1.43 (0.78–2.62)	
Winter	134	17 (12.7)	1	1	

1 = reference group, Not applicable = children < 5 years, spring = September to November, winter = December to February, autumn = March to May, summer = June to August

* = statistically significant (p-value < 0.05), COR (95%CI) = crudes odds ratio at 95% confidence interval, AOR (95%CI) = adjusted odds ratio at 95% confidence interval, MP-value = multivariate p value, anti-DENV IgM+ = anti-dengue virus immunoglobulin M positive

Univariate logistic regression analysis of the investigated variables showed statistically significant associations (p-value < 0.05) with the prevalence of anti-DENV IgM-/G+ serostatus was observed with age, gender, residence, occupational status and the study area. In multivariate logistic regression analysis, gender, residence, occupational status, and the study area were significantly associated with the prevalence of anti-DENV IgM-/G+ serostatus. Males had 2 times (AOR = 2.05; 95% CI = 1.14–3.69) higher prevalence of anti-DENV IgM-/G+ serostatus than females. Urban residents had 2.3 times (AOR = 2.31; 95% CI = 1.38–3.87) higher prevalence of anti-DENV IgM-/G+ serostatus than rural residents. Farmers had 2.8 times (AOR = 2.81; 95% CI = 1.23–6.41) higher prevalence of anti-DENV IgM-/G+ serostatus than students. Study participants who were visited Metema hospital had 1.7 times (AOR = 1.75; 95%CI = 1.05–2.89) higher prevalence of anti-DENV IgM-/G+ serostatus than those visited Humera hospital (Table 5).

**Table 5:** Factors associated with the prevalence of anti-DENV IgM-/G+ serostatus among the study participants, from March 2016 to May 2017.

Variable	Total No. tested (N = 600)	Anti-DENV IgM-/G+	A univariate analysis	Multivariate analysis	MP-value
		N (%) (N = 86)	COR (95%CI)	AOR (95%CI)	
**Age**					
≤ 14	116	8 (6.9)	1	1	
15–29	302	49 (16.2)	2.61 (1.19–5.70)*	1.24 (0.47–3.25)	0.654
30–44	114	21 (18.4)	3.04 (1.29–7.20)*	1.27 (0.43–3.74)	0.654
≥ 45	68	8 (11.8)	1.80 (0.64–5.04)	0.93 (0.27–3.16)	
**Gender**					
Male	394	69 (17.5)	2.36 (1.34–4.13)*	2.05 (1.14–3.69)*	0.016
Female	206	17 (8.3)	1	1	
**Residence**					
Urban	316	56 (17.7)	1.82 (1.13–2.93)*	2.31 (1.38–3.87)*	0.001
Rural	284	30 (10.6)	1	1	
**Marital status**					
Married	255	36 (14.1)	1		
Single	332	48 (14.5)	1.02 (0.64–1.64)		
Divorced	13	2 (15.4)	1.10 (0.23–5.19)		
**Occupation**					
Farmer	301	56 (18.6)	3.20 (1.62–6.29)*	2.81 (1.23–6.41)*	0.014
Not applicable	14	1 (7.1)	1.07 (0.12–9.01)	1.38 (0.15–12.74)	
Shopkeeper	48	8 (16.7)	2.8 (1.05–7.42)*	2.60 (0.88–7.70)	
Government employee	34	4 (11.8)	1.86 (0.55–6.25)	1.36 (0.37–5.05)	
Day laborer	38	6 (15.8)	2.62 (0.90–7.61)	2.10 (0.65–6.76)	
Student	165	11 (6.7)	1	1	
**Educational status**					
Illiterate	184	27 (14.7)	1.89 (0.42–8.51)		
Only read and write	145	21 (14.5)	1.86 (0.40–8.51)		
Elementary school	170	27 (15.9)	2.07 (0.46–9.35)		
Secondary	77	9 (11.7)	1.45 (0.29–7.25)		
Higher education	24	2 (8.3)	1		
**Study area**					
Humera	320	34 (10.6)	1	1	
Metema	280	52 (18.6)	1.91 (1.20–3.05)*	1.75 (1.05–2.89)*	0.029
**Season**					
Summer	77	16 (20.8)	1.93 (0.90–4.13)		
Spring	56	13 (23.2)	2.23 (0.99–5.01)		
Autumn	333	41 (12.3)	1.03 (0.55–1.91)		
Winter	134	16 (11.9)	1		

1 = reference group, Not applicable = children < 5 years, spring = September to November, winter = December to February, autumn = March to May, summer = June to August

* = statistically significant (p-value < 0.05), COR (95%CI) = crudes odds ratio at 95% confidence interval, AOR (95%CI) = adjusted odds ratio at 95% confidence interval, MP-value = multivariate p value, anti-DENV IgM-/G+ = anti-dengue virus immunoglobulin M negative but immunoglobulin G positive.

### Risk factors associated with the prevalence of anti-DENV antibodies

In a univariate analysis lack of mosquito net use, the presence of uncovered water storage either indoor or outdoor and history of travel abroad were significantly associated with the prevalence of anti-DENV IgM seropositivity. However, use of mosquito repellant, indoor insecticidal spraying, keeping the animal at home, slaughtering an animal, seasonal migrant laborer and a place where to sleep were not significantly associated (p-value > 0.05). In multivariate analysis, history of travel to abroad was not significantly associated with the prevalence of anti-DENV IgM seropositivity while lacking use of a mosquito net and the presence of uncovered water storage either indoor or outdoor remained significantly associated. Lack of mosquito net use was 1.7 times (AOR = 1.75; 95% CI = 1.00–3.06) higher risk of DENV infection than those who have used it. Those study participants who stored water without cover either indoor or outdoor had 1.6 times (AOR **=** 1.60; 95% CI = 1.05–2.43) higher risk of DENV infection than those who didn’t store it (Table 6).

**Table 6:** Risk factors associated with the prevalence of anti-DENV IgM seropositivity among the study participants, from March 2016 to May 2017.

Variable	Total No. participants(N = 600)	Anti-DENV IgM+	A univariate analysis	Multivariate analysis	MP-value
		N (%) (N = 114)	COR (95%CI)	AOR (95%CI)	
**Mosquito net use**					
Yes	520	91 (17.5)	1	1	
No	80	23 (28.7)	1.9 (1.11–3.24)*	1.75 (1.00–3.06)*	0.049
**Use mosquito repellent**					
Yes	225	40 (17.8)	1		
No	375	74 (19.7)	1.13 (0.74–1.74)		
**Indoor insecticidal spraying**					
Yes	197	34 (17.3)	1		
No	403	80 (19.9)	1.18 (0.76–1.85)		
**Keeping animal at home**					
Yes	337	68 (20.2)	1.19 (0.78–1.80)		
No	263	46 (17.5)	1		
**Indoor or outdoor water storage**					
Yes	261	61 (23.4)	1.64 (1.09–2.48)*	1.60 (1.05–2.43)*	0.026
No	339	53 (15.6)	1	1	
**Animal slaughter**					
Yes	161	34 (21.1)	1.20 (0.76–1.88)		
No	439	80 (18.2)	1		
**Seasonal migrant laborer**					
Yes	65	17 (26.2)	1.59 (0.88–2.90)		
No	535	97 (18.1)	1		
**History of travel to abroad**					
Sudan	44	14 (31.8)	2.12 (1.08–4.16)*	1.62 (0.80–3.30)	0.177
No	556	100 (18)	1		
**Usually, sleep**					
Inside the home	294	61 (20.7)	1.25 (0.83–1.88)		
Outside the home	306	53 (17.3)	1		

1 = reference group

* = statistically significant (p-value < 0.05), COR (95%CI) = crudes odds ratio at 95% confidence interval, AOR (95%CI) = adjusted odds ratio at 95% confidence interval, MP-value = multivariate p value, anti-DENV IgM+ = anti-dengue virus Immunoglobulin M positive.

In the univariate and multivariate analysis, lack of mosquito net use and the presence of uncovered water storage either indoor or outdoor were significantly associated with the prevalence of anti-DENV IgM-/G+ serostatus. Whereas, the use of mosquito repellent, indoor insecticidal spraying, keeping the animal at home, slaughtering an animal, seasonal migrant laborer, history of travel to abroad and a place where to sleep were not significantly associated with the prevalence of anti-DENV IgM-/G+ serostatus (Table 7).

**Table 7:** Risk factors associated with the prevalence of anti-DENV IgM-/G+ serostatus among the study participants, from March 2016 to May 2017.

Variable	Total No. participants (N = 600)	Anti-DENV IgM-/G+	A univariate analysis	Multivariate analysis	MP-value
		N (%) (N = 86)	COR (95%CI)	AOR (95%CI)	
**Mosquito net use**					
Yes	520	67 (12.9)	1	1	
No	80	19 (23.8)	2.10 (1.18–3.74)*	2.16 (1.20–3.88)*	0.009
**Use mosquito repellent**					
Yes	225	35 (15.6)	1.17 (0.73–1.86)		
No	375	51 (13.6)	1		
**Indoor insecticidal spraying**					
Yes	197	30 (15.2)	1.11 (0.68–1.80)		
No	403	56 (13.9)	1		
**Keeping animal at home**					
Yes	337	47 (13.9)	1		
No	263	39 (14.8)	1.07 (0.67–1.70)		
**Indoor or outdoor water storage**					
Yes	261	52 (19.9)	2.23 (1.40–3.55)*	2.26 (1.41–3.62)*	0.001
No	339	34 (10)	1	1	
**Animal slaughter**					
Yes	161	27 (16.8)	1.29 (0.79–2.13)		
No	439	59 (13.4)	1		
**Seasonal migrant laborer**					
Yes	65	13 (20)	1.58 (0.82–3.04)		
No	535	73 (13.6)	1		
**History of travel to abroad**					
Sudan	44	9 (20.5)	1.60 (0.74–3.45)		
No	556	77 (13.8)	1		
**Usually, sleep**					
Inside the home	294	46 (15.6)	1.23 (0.78–1.94)		
Outside the home	306	40 (13.1)	1		

1 = reference group

* = statistically significant (p-value < 0.05), COR (95%CI) = crudes odds ratio at 95% confidence interval, AOR (95%CI) = adjusted odds ratio at 95% confidence interval, MP-value = multivariate p value, anti-DENV IgM-/G+ = anti-dengue virus Immunoglobulin M negative but Immunoglobulin G positive.

## Discussion

This is the first health institution based study that provides evidence on seroprevalence of DENV infection into two towns of Northwest Ethiopia. The results of this study showed a seroprevalence of DENV infection of 40% in Metema and 27.5% in Humera. Several factors favor transmission of DENV in the study areas. Such as the existing climatic conditions (i.e., high temperature), providing the optimal environmental and biological circumstances for vector mosquito breeding and reproduction, and also increased urbanization in the study areas might favor the emergence and survival of DENV infected *Aedes* mosquitoes [16]. The variations of seroprevalence between the study areas might be due to differences in virus circulation inhabitants of these areas which correspond to the area with low virus circulation might have had low transmission of the virus [25]. Dengue is a preventable disease that causes significant morbidity and mortality in most tropical and sub-tropical countries of the world [26–29]. The evidence generated here are essential not only for patient management but also for undertaking early prevention and control interventions; such as reduction of mosquito breeding in household water containers using larvicides, or elimination of discarded containers, and control of adult mosquitoes by spraying insecticides or prevent them from biting using repellent [2].

The overall seroprevalence of DENV infection in the present study was 33.3%, which is lower than the recent study in Dire Dawa Ethiopia, 56.8% [12]. The difference of the prevalence rates between the two studies might be due to variations in dengue positive considerations; the previous study considered dengue positives either by ELISA/PCR while the current study considered dengue positives only by ELISA test. A similar result was reported in Eritrea 33.3% [9]. However, this result is higher than the findings in Tanzania 7.7% [30], in Kenya, 12.5% [10], in Djibouti, 21.8% [11], in the Northern Province of Sudan, 24% [8], and lower than the study in Thailand, 51.5% [31], and in Kassala, Eastern Sudan, 71.7% [7]. The variations might be due to the differences in the environmental factors such as temperature, rainfall, and humidity which affect dengue transmission [32]. Despite one-third of the study participants had antibody against DENV infection, dengue was underrecognized and underreported in Ethiopia, which is in line with an earlier report in Africa [6]. The possible reasons for underrecognition of the DENV infection in Ethiopia might be due to the similarity of most of the clinical presentations of DENV infection with the other febrile illnesses and the lack of documented previous data on the occurrence of DENV which provides awareness of the disease among health providers.

The overall prevalence of anti-DENV IgM seropositivity was 19%, while anti-DENV IgM+/G- serostatus was 12.3%, likely indicates recent infection with DENV. Since IgM against the DENV infection can be usually detected after the first 5 days of infection and peaks approximately 14 days after the onset of disease and may persist up to 3 months [33, 34]. However, the possibility that as the IgM antibodies remain negative for the first few days, and also the IgM reactivity was non-specific; is thus cross-reactive due to infection with another flavivirus cannot be excluded [35]. The prevalence of anti-DENV IgM+/G+ serostatus was 6.7%. This might indicate that it is likely that the person became infected with DENV within recent weeks; as IgM response is usually detected after the 5 days of illness, and followed by IgG response [34]. The prevalence of anti-DENV IgM-/G+ serostatus was 14.3%, might indicate probably past infection with DENV. This seroprevalence may be overestimated by false positive results; since we could not collect paired samples, the presence of anti-DENV IgM-/G+ might be confounded with the issue of cross-reactivity of other anti-flavivirus antibodies [35]. However, recently in Dire Dawa Ethiopia, of serum samples tested for arboviruses, it was DENV which has been reported [12], this suggests that cross-reactivity with antibodies from other arboviruses might be less likely in the present study.

According to 2009 WHO-TDR classification scheme [2], suggested dengue cases have been classified into two categories, dengue without warning signs and dengue with warning signs. Similar findings were reported elsewhere [36], but other studies reported previously three of the categories including severe dengue cases [37, 38]. In this study severe dengue cases were not noticed, this might be due to probably primary infection with any one of the four DENV serotypes which are usually associated with mild disease. Since it is a subsequent infection of DENV with a second heterologous serotype which increases the severity of dengue [39, 40]. The immune basis for this hypothesis suggests that antibodies developed during the primary infection instead of mediating viral clearance, assist the new virus in infecting host macrophages, a phenomena known as antibody-dependent enhancement [41].

In this study, anti-DENV IgG seropositivity was observed in every month of the year. This suggests potential endemicity of DENV infection; as studies illustrated IgG against the DENV infection might have persisted for several decades [42]. Previous vaccination against yellow fever may render the anti-DENV IgG ELISA test less specific because of cross-reaction [43]. However, in the present study, none of the study participants had been vaccinated against yellow fever; hence natural antibody response which occurs after anti-yellow fever vaccination is unlikely.

In the present study, the analysis of the seasonal variation was significantly associated with the prevalence of anti-DENV IgM seropositivity but not with anti-DENV IgM-/G+ serostatus. The highest prevalence of anti-DENV IgM seropositivity observed during summer (June to August), followed by spring (September to November), and indicated the presence of high viral transmission during monsoon and post-monsoon periods. These findings are consistent with the other studies [44, 45]. The study suggests effective vector control and preventive measures to be implemented during water stagnation periods after the initial bouts of rainfall and at the end of monsoon which corresponds to the breeding time of the dengue vectors [46].

Findings from this study showed the prevalence of anti-DENV IgM-/G+ serostatus was significantly higher in males than in females, which is in agreement with the study elsewhere [47] while others reported no significant difference between genders [48]. Although the prevalence of anti-DENV IgM seropositivity was not significantly associated with gender, its prevalence in males was higher than in females, which is in line with other studies [9, 47, 49]. The higher proportion of anti-DENV IgM seropositivity in males might be associated with DENV vectors, *Ae*. *aegypti* and *Ae*. *albopictus* breeding sites which are more abundant in outdoors than indoors [50]. In the study areas, it is assumed that males might have greater involvement in outdoor activities than females, and thus possibly increase exposure to day feeding *Ae*. *aegypti* mosquito, which is the main vector of DENV [2]. Concerning study area, the prevalence of anti-DENV IgM seropositivity was not significantly associated while anti-DENV IgM-/G+ serostatus was significantly higher in Metema than Humera. This is indicative of an intense and long-lasting exposure of population who lived in Metema area to DENV infection risks.

With regards to age groups, studies elsewhere reported that children were the most dengue affected subpopulation [51, 52]. However, in this study anti-DENV, seropositivity was noticed in all age groups with none significantly higher seroprevalence that was observed in age groups of 30–45 years old. These findings are consistent with other studies [12, 53]. This may be due to the increased possibility of being exposed to dengue vector bite as age increased and becoming seropositive for DENV infection over the lifetime of the individual [42, 54]. Educational status of the participants in this study was not significantly associated with anti-DENV antibodies seropositivity and this is consistent with another study [48]. This could be due to the fact that previously, the disease is not known in the areas and even not included under the lists of nationally reportable diseases which daunt to use any preventive measures of DENV infection.

In this study, those individuals who reside in urban areas were more affected than those in the rural areas. This is in agreement with the studies elsewhere [55, 56], and this is also consistent with the current knowledge on the dengue main vector, *Ae*. *aegypti* increases in urban environments in that it breeds mainly in the artificial containers often used in urban water collection [16]. There was also a significant association between occupations of the study participants with anti-DENV antibodies seropositivity. Farmers were more affected than other groups with a different occupation. Since the vector is active at daytime and mostly prefers to rest in the shade of trees or nearby buildings. Because of the high temperature in our study areas, most of the time, farmers take rest in the shade of trees which increase exposure to day feeding outdoor *Aedes* mosquitoes.

In the present study, participants who had lack of mosquito net use during sleeping were found to be more at risk of getting DENV infection. This could be due to sleeping without mosquito net increases the contact between mosquitoes and humans, and thus increase transmission of mosquito-borne diseases including dengue [2]. However, study elsewhere showed that no significant difference between mosquito net users and none users [48]. The variations might be probably differences in the quality of mosquito net and the frequency of impregnating nets with chemicals. The presence of stagnant water either indoor or outdoor was identified as the risk factor of dengue, which is consistent with the other studies in elsewhere [57, 58]. This finding may be explained by the fact that long-term storage of water in opened containers favors breeding of the mosquito vector. This results in an increase in dengue cases, and thus frequent emptying of long-term stagnant water storage inside and outside the house is recommended to reduce mosquito breeding sites [2].

Although this is the first attempt to study a seroprevalence and risk factors associated with DENV infection in Northwest Ethiopia, the study has several limitations. A comparison of the acute serum with the convalescent serum from the same patients was not done due to the nature of a cross-sectional study design. The study was conducted among febrile patients who were attending only health institutions that may not necessarily reflect the true seroprevalence at the community level where some mild or asymptomatic infection might occur. There was no information on the median duration of fever for enrolled subjects, and also RT-PCR and plaque reduction neutralization tests (PRNT) were not done due to lack of facilities. These could be done in the future for more accurate estimation of overall prevalence and for identification of circulating serotypes and genotypes. Moreover, this study has also limitations including the presence of a possibility of false negative dengue cases due to IgM antibodies remains negative for the first few days of fever and thus we cannot state that these subjects with negative IgM did not have acute dengue. There is also a possibility of some false positive cases due to cross-reactivity of other anti-flavivirus antibodies with DENV. Despite these limitations, this preliminary study ultimately provides the first baseline data on seroprevalence of DENV infection and associated risk factors in the country.

In conclusion, one-third of patients who presented with fever presumed of dengue had antibodies against DENV infection; this should alert all concerned parties within the health sectors. The prevalence of anti-DENV IgM seropositivity was significantly associated with residence, occupation and season. In addition to this, the presence of uncovered water storage either indoor or outdoor was identified as the risk factor of DENV infections. Therefore, we recommend that the prevention strategies and control measures should be designed in the country considering the risk factors identified by this study. Moreover, nationwide surveillance should be done at large to include the dengue in the differential diagnosis of all febrile cases, in Ethiopia.

## Supporting information

S1 STROBE Checklist (DOC)
