# Supplementary material for: A serologic study of dengue in northwest Ethiopia: Suggesting preventive and control measures
Source: PLoS Negl Trop Dis. 2018 May 31;12(5):e0006430. doi: 10.1371/journal.pntd.0006430 (PMC5978788; doi:10.1371/journal.pntd.0006430)
Supplement: S1 STROBE Checklist — (DOC) [file pntd.0006430.s001.doc]

**Supporting information files**

STROBE Statement—Checklist of items that should be included in reports of ***cross-sectional studies***

|  | Item No | Recommendation |
| --- | --- | --- |
| **Title and abstract** | 1 | (*a*) Indicate the study’s design with a commonly used term in the title or the abstract |
|  |  | **The study is a cross-sectional hospital based study using none probability (convenient) sampling design.** |
|  |  | (*b*) Provide in the abstract an informative and balanced summary of what was done and what was found.  **Our study detected anti-dengue virus IgG and IgM in human blood samples in both study areas of Northwest Ethiopia for the first time. In addition, the findings identified the presence of open water and not uses of mosquito net as risk factors for dengue virus infection.** |
| Introduction | | |
| Background/rationale | 2 | Explain the scientific background and rationale for the investigation being reported  **Although dengue virus infection has been confirmed recently in Ethiopia, data are scarce in the country. Particularly, in Northwest Ethiopia, there is no documented data.** |
| Objectives | 3 | State-specific objectives, including any prespecified hypotheses  **The study was conducted to document seroprevalence and associated risk factors of dengue virus infection in the study areas for the first time which helps to improve the management of the patents.** |
| Methods | | |
| Study design | 4 | Present key elements of study design early in the paper  **A cross-sectional study was conducted to detect the presence/absence of dengue virus infection in Metema and Humera hospitals, using convenient sampling method.** |
| Setting | 5 | Describe the setting, locations, and relevant dates, including periods of recruitment, exposure, follow-up, and data collection  **The study has been conducted in Northwest Ethiopia from March 2016 to May 2017.** |
| Participants | 6 | (*a*) Give the eligibility criteria and the sources and methods of selection of participants  **All dengue suspected febrile patients who were visiting health institutions were included in the study.** |
| Variables | 7 | Clearly define all outcomes, exposures, predictors, potential confounders, and effect modifiers. Give diagnostic criteria, if applicable  **Outcome: anti-dengue IgG and/ IgM. Predictors: Residence and occupational statuses, seasonal variation, Not use of mosquito nets and presence of opened water.** |
| Data sources/ measurement | 8* | For each variable of interest, give sources of data and details of methods of assessment (measurement). Describe comparability of assessment methods if there is more than one group  **Information was obtained from each study participant. Blood samples were collected from all volunteers during a hospital visit. Samples were tested by experience laboratory technologist.** |
| Bias | 9 | Describe any efforts to address potential sources of bias  **We included all dengue suspected patients who were visiting health institutions during the study period. This prevents bias introduction.** |
| Study size | 10 | Explain how the study size was arrived at  **Since there is no any previous study in the areas, due to this we used 50% prevalence and calculated using single proportion formula to determine sample size.** |
| Quantitative variables | 11 | Explain how quantitative variables were handled in the analyses. If applicable, describe which groupings were chosen and why  **No groupings were chosen** |
| Statistical methods | 12 | (*a*) Describe all statistical methods, including those used to control for confounding  **Data were analyzed using a univariate logistic regression. Those associated variables p < 0.25 with seropositivity in univariate analysis, we then used in a multivariate logistic regression.** |
|  |  | (*b*) Describe any methods used to examine subgroups and interactions  **No interactions were tested** |
|  |  | (*c*) Explain how missing data were addressed  **No missing data present** |
|  |  | (*d*) If applicable, describe analytical methods taking account of sampling strategy  **Not applicable** |
|  |  | (*e*) Describe any sensitivity analyses  **Not applicable** |
| Results | | |
| Participants | 13* | (a) Report numbers of individuals at each stage of study—eg numbers potentially eligible, examined for eligibility, confirmed eligible, included in the study, completing follow-up, and analyzed  **The study has been conducted from March 2016 to May 2017 in Both study areas (Metema and Humara). 320 and 280 samples were collected in Humera and Metema hospitals respectively. A total of 600 serological results were available.** |
|  |  | (b) Give reasons for non-participation at each stage  **All of the study participants were volunteer to participate** |
|  |  | (c) Consider use of a flow diagram  **Not considered** |
| Descriptive data | 14* | (a) Give characteristics of study participants (eg demographic, clinical, social) and information on exposures and potential confounders  **Better to look at the table.** |
|  |  | (b) Indicate the number of participants with missing data for each variable of interest  **None** |
| Outcome data | 15* | Report numbers of outcome events or summary measures  **Out of the 600 samples tested, the overall seroprevalence against DENV infection was 33.3% while the seroprevalence by the study area was 40% at Metema and 27.5% at Humara. The total IgM and IgG seroprevalence against DENV was found 19% and 21% respectively. Of those positives for dengue IgM and IgG, 6.7% were found to be positive for both IgM and IgG antibodies.** |
| Main results | 16 | (*a*) Give unadjusted estimates and, if applicable, confounder-adjusted estimates and their precision (eg, 95% confidence interval). Make clear which confounders were adjusted for and why they were included  **Better to look at the table.** |
|  |  | (*b*) Report category boundaries when continuous variables were categorized  **Not applicable** |
|  |  | (*c*) If relevant, consider translating estimates of relative risk into absolute risk for a meaningful time period  **Not applicable** |
| Other Analyses | 17 | Report other analyses done—eg analyses of subgroups and interactions and sensitivity analyses  **Not applicable** |
| Discussion | | |
| Key results | 18 | Summarise key results with reference to study objectives  **Anti-dengue seroprevalence of 40% and 27.5%, in Metema and Humara hospital, respectively were found. Residence and occupational statuses were significantly associated with both the dengue IgM and only dengue IgG prevalence. The seasonal variation was significantly associated with the dengue IgM prevalence but not with only dengue IgG prevalence. The majority of the dengue IgM prevalence was found in the summer and spring, with a peak in the month of August. The presence of opened water either indoor or outdoor and never uses of mosquito net were identified as risk factors.** |
| Limitations | 19 | Discuss limitations of the study, taking into account sources of potential bias or imprecision. Discuss both direction and magnitude of any potential bias  **This study has some limitations, including the fact that serological tests have. There is the possibility of false negative dengue cases due to IgM antibody remains negative for the first few days of fever and also there is the possibility of some false positive cases due to cross-reactivity of other flaviviral infections with dengue. However, during a study participant’s enrollment period, we were critically considered dengue suspected cases based on 2009 WHO criteria and also we were used ELISA which is a serologic gold standard diagnostic method, all these, increases the reliability of our results.** |
| Interpretation | 20 | Give a cautious overall interpretation of results considering objectives, limitations, a multiplicity of analyses, results from similar studies, and other relevant evidence  **Antibodies (IgG and IgM) against dengue virus infection were observed in Northwest Ethiopia. In addition, associated and risk factors were identified. All these help to deploy interventions in the country.** |
| Generalisability | 21 | Discuss the generalisability (external validity) of the study results  **Unrecognized dengue transmission has existed in Northwest Ethiopia.** |
| Other information | | |
| Funding | 22 | Give the source of funding and the role of the funders for the present study and, if applicable, for the original study on which the present article is based  **This study was conducted with the support of the University of Gondar and the Ministry of Health of Ethiopia through Armauer Hansen Research Institute. The funders had no role in study design, data collection, and analysis, decision to publish, or preparation of the manuscript** |

*Give information separately for exposed and unexposed groups.

**Note:** An Explanation and Elaboration article discusses each checklist item and gives methodological background and published examples of transparent reporting. The STROBE checklist is best used in conjunction with this article (freely available on the Web sites of PLoS Medicine at http://www.plosmedicine.org/, Annals of Internal Medicine at http://www.annals.org/, and Epidemiology at http://www.epidem.com/). Information on the STROBE Initiative is available at www.strobe-statement.org.
